# Linkage mapping, comparative genome analysis, and QTL detection for growth in a non-model teleost, the meagre *Argyrosomus regius*, using ddRAD sequencing

**DOI:** 10.1038/s41598-022-09289-4

**Published:** 2022-03-29

**Authors:** O. Nousias, S. Oikonomou, T. Manousaki, V. Papadogiannis, N. Angelova, D. Tsaparis, A. Tsakogiannis, N. Duncan, A. Estevez, K. Tzokas, M. Pavlidis, D. Chatziplis, C. S. Tsigenopoulos

**Affiliations:** 1 Institute of Marine Biology, Biotechnology and Aquaculture (IMBBC), Hellenic Centre for Marine Research (HCMR), Heraklion, Greece; 2 Department of Biology, University of Crete, Heraklion, Greece; 3 Department of Agriculture, International Hellenic University (IHU), Thessaloniki, Greece; 4 IRTA Institute of Agrifood Research and Technology, Barcelona, Spain; 5 Andromeda S.A., Agios Vasilios, Rion, Greece

**Keywords:** Genetic linkage study, Comparative genomics, Genetics, Quantitative trait

## Abstract

Meagre (*Argyrosomus regius*), is a benthopelagic species rapidly emerging in aquaculture, due to its low food to biomass conversion rate, good fillet yield and ease of production. Tracing a species genomic background along with describing the genetic basis of important traits can greatly influence both conservation strategies and production perspectives. In this study, we employed ddRAD sequencing of 266 fish from six F1 meagre families, to construct a high-density genetic map comprising 4529 polymorphic SNP markers. The QTL mapping analysis provided a genomic appreciation for the weight trait identifying a statistically significant QTL on linkage group 15 (LG15). The comparative genomics analysis with six teleost species revealed an evolutionarily conserved karyotype structure. The synteny observed, verified the already well-known fusion events of the three-spine stickleback genome, reinforced the evidence of reduced evolutionary distance of Sciaenids with the Sparidae family, reflected the evolutionary proximity with *Dicentrarchus labrax*, traced several putative chromosomal rearrangements and a prominent putative fusion event in meagre’s LG17. This study presents novel elements concerning the genome evolutionary history of a non-model teleost species recently adopted in aquaculture, starts to unravel the genetic basis of the species growth-related traits, and provides a high-density genetic map as a tool that can help to further establish meagre as a valuable resource for research and production.

## Introduction

The Sciaenidae family, of the diverse order Perciformes, consists of approximately 300 species^1^, 70 genera^2^, are known for the production of throbbing sounds during the mating period. Sciaenids are found worldwide, both in fresh and marine waters, and are typically benthopelagic carnivores. Three Sciaenids are considered to have an established aquaculture production, the large yellow croaker (*Larimichthys crocea*)^3^, the yellow croaker (*Larimichthys polyactis*)^4^, and the red drum (*Sciaenops ocellatus*)^5^; the Japanese meagre or mulloway (*Argyrosomus japonicus*), is mainly farmed in Australia^6^ and lately in South Africa. The meagre (*Argyrosomus regius*) is mainly found in the inshore waters where the sea is relatively shallow compared to the open ocean. The geographical distribution of meagre spans from Norway to West Africa in the Atlantic Ocean, and to the east includes the Mediterranean and Black Sea. The phenotypic and life history traits, such as high growth rate and ease of larval rearing^7–9^ have facilitated the selection and development of commercial culture of the species as a food fish and aquaculture production of meagre in the Mediterranean basin has risen from 30 tonnes in 1997 to 37,526 tonnes in 2019 (fao.org). Marker assisted selection and genomic selection, in aquaculture has and is still transforming the industry, providing genetic information and linkage maps for approximately 40 species since the 1990s^10–12^. The availability of genetic maps, is a key factor in the investigation of genome evolution, linking genotypes to phenotypes for traits of interest, and improving the genome assembly^13–15^. The de novo discovery and simultaneous scoring of thousands of single nucleotide polymorphisms (SNPs), is a new means of characterizing the genomes of non-model species. These genome-reduction screening techniques provide alternative approaches for these different applications. The analysis, RAD-Seq^16^ allows the identification of many thousands of SNPs, but requires considerable sequencing effort per individual. Variants of this method, e.g., Genotyping-by-Sequencing^17^, ddRAD^18^, 2bRAD^19^, ezRAD^20^, can limit the extent of marker discovery, thereby allowing sequencing of a greater number of individuals. Genetic linkage maps using genotyping by sequencing approaches have already been produced for numerous fishes, such as the spotted gar (*Lepisosteus oculatus*)^21^, Midas cichlid (*Amphilophus* spp.)^22^, gudgeon (genus *Gnathopogon*)^23^, blind cavefish (*Astyanax mexicanus*)^24^, Atlantic halibut (*Hippoglossus hippoglossus*)^25^, Common Pandora (*Pagellus erythrinus*)^26^, Nile tilapia (*Oreochromis niloticus*)^27^, orange-spotted grouper (*Epinephelus coioides*)^28^, Japanese eel (*Anguilla japonica*)^29^, and platyfish (*Xiphophorus maculatus*)^30^, among others^11^. Furthermore, high-density genetic maps have been constructed for several members of the Sciaenidae family, e.g. yellow croaker (*Larimichthys crocea*)^31^, small yellow croaker (*Larimichthys polyactis*)^32^, and red drum (*Sciaenops ocellatus*)^33^.

It is well-known that teleost have undergone three rounds of whole genome duplication (WGD)^34^ with the third round having occurred ~ 370 million years ago (MYA)^35^. The common ancestor of most teleost, ~ 50 MY after the last duplication event, underwent eight major inter-chromosomal rearrangements (2 fissions, 4 fusions and 2 translocations), which themselves lead to an ancestral karyotype of 24 chromosomes^36^. Notedly, the medaka (*Oryzias latipes*) genome seemingly preserved the ancestral karyotype without undergoing major inter-chromosomal rearrangements for more than ~ 300 MY^37^. Comparative analysis of numerous teleost published genomes revealed that they underwent inter-chromosomal rearrangements after speciation from a common ancestor^38,39^, and that they have fewer or more chromosomes than medaka. However, more research is needed to determine how common the preservation of the ancestral karyotype, from which most teleost originated, is amongst present day teleost.

To improve aquaculture production for meagre, it is important to identify markers linked to economically important traits. Quantitative Trait Loci (QTL) analyses enable us to find genetic markers associated with the variation for production traits and their candidate genes, which can be utilized for breeding programs. To date, several QTL have been identified concerning growth-related traits in three members of the Sciaenidae family, yellow croaker, small yellow croaker, and mulloway^31,32,40^. In this study, we performed ddRAD sequencing for all progeny and parents of six meagre families. After filtering, we constructed the first high-density genetic map for meagre, and we used the markers on the map to examine the synteny between meagre and six other fish species, including the stickleback (*Gasterosteus aculeatus*), medaka (*Oryzias latipes*), European seabass (*Dicentrarchus labrax*), yellow croaker (*Larimichthys crocea*), gilthead seabream (*Sparus aurata*), and tilapia (*Oreochromis niloticus*), and trace putative rearrangements in the meagre genome. Finally, by performing a QTL analysis in meagre, a statistically significant genetic locus linked to bodyweight was identified; this locus is highly similar to coding sequences of the *eys* gene, which have been reported to be associated with growth in three species^41^.

## Results

### SNP calling by mapping ddRAD sequencing reads to the reference genome

From a total of 688 × 10^6^ primary BAM records aligned against the meagre genome sequence developed in-house (Papadogiannis et al., in preparation), 577 × 10^6^ (87%) passed the quality control using a phred score Q = 20, and 281 × 10^6^ were complementary reads in 3′–5′ direction. Sequences excluded were some 26 × 10^6^ (4%) not sufficiently aligned to the genome, some 51 × 10^6^ (7.8%) partially aligned, and 3 × 10^6^ (0.4%) not aligned with the genome. The RAD loci derived from gstacks^42^ were 316,425, with an average base length of 353.1 (sd = 106.7). The coverage for each locus was 43.3 × on average. The populations stacks module^42^ excluded 294,953 and kept 21,472 sites, which contained 9710 SNPs. The mean number of base pairs (bp) for each RAD locus was 414.67 bp (sd = 0.73).

### High-density genetic linkage mapping

**Figure 1:**
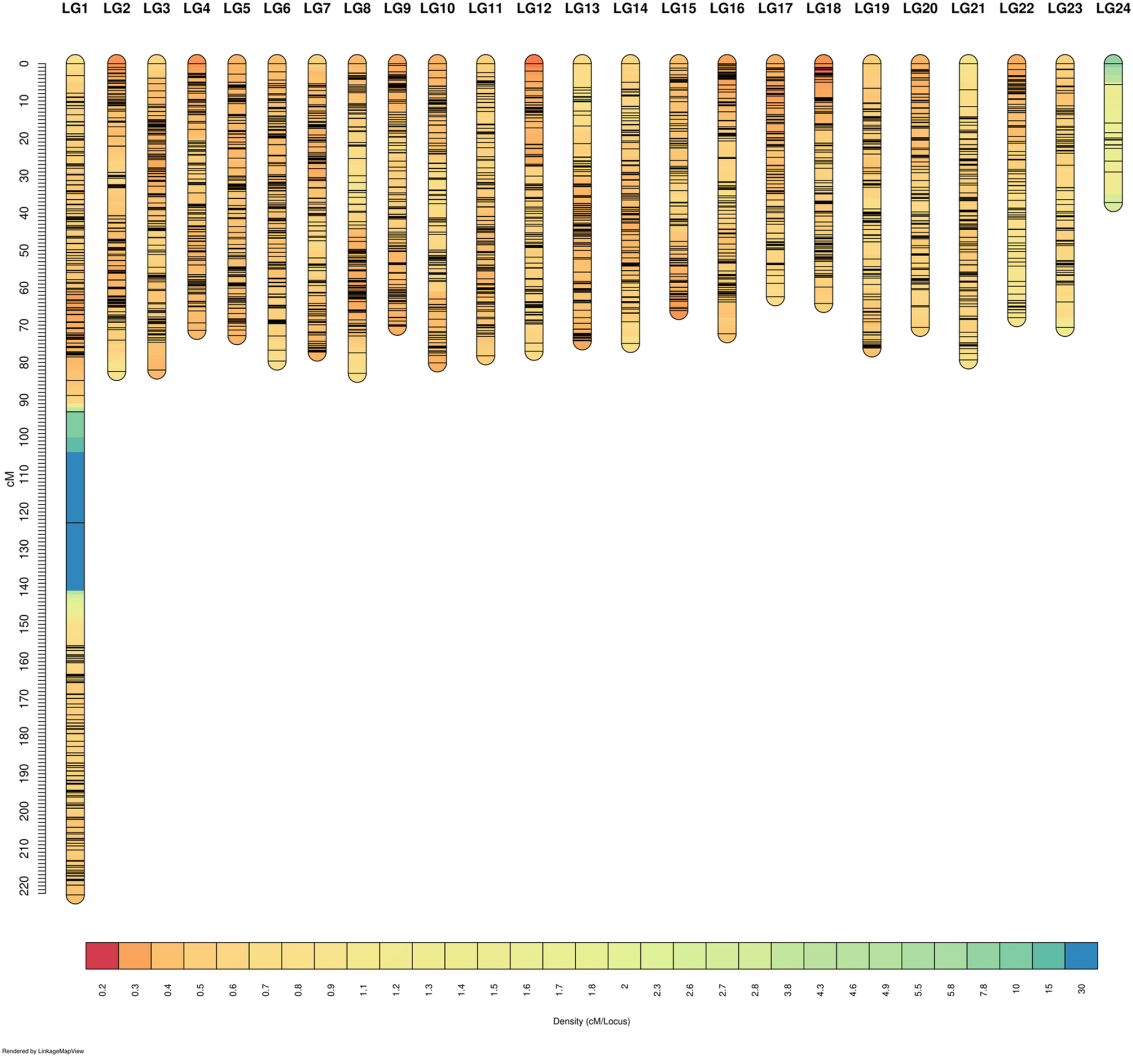
Genetic map originating from a subset of 70% shared RAD loci among individuals. The colouring of the marker intervals corresponds to a decrease in density, from red to blue.

Out of the 9710 RAD markers, after the quality control using the 2% permissible error limit per RAD locus according to Mendel inheritance, 5988 markers remained. Then, 4529 markers were clustered in the 24 linkage groups of the genetic map, while 1459 markers remained unlinked; the composition of the genetic linkage map is given in Table S2. The LOD score used was 4.80 and the SNPs that passed the data tolerance threshold (0.001) were included in the linkage groups; the command lines used are from Oikonomou et al.^43^. The smallest linkage group (here after referred to as LG) consists of 20 RAD markers (LG24) and the largest (LG1) of 396, while the average inter-marker distance on the map was 0.4 cM and the total map length 1158 cM. The LG5 showed the smallest average distance between markers (0.33 cM), and LG24 showed the largest (1.86). The largest distance pairs of markers were found in LG1, with two gaps of 31 and 29 cM. A second map was constructed, from a subset of SNP markers with higher coverage (see “Methods”), for evaluation purposes (Fig. S8). The second map, resulted in a smaller LG1 by 30 cM with the remaining LGs having similar lengths and intervals, as in the main genetic map that is presented in this study. Additionally, the male specific map totalled 1899 cM and the female one 1987 cM, showing high similarity between them (Table S1), resulting in LGs with approximately the same length on average and intervals, while in total they were both equally longer than the map presented here (Fig. 1, Table S2) (Supplementary data file 1).

### Comparative genomics

**Figure 2:**
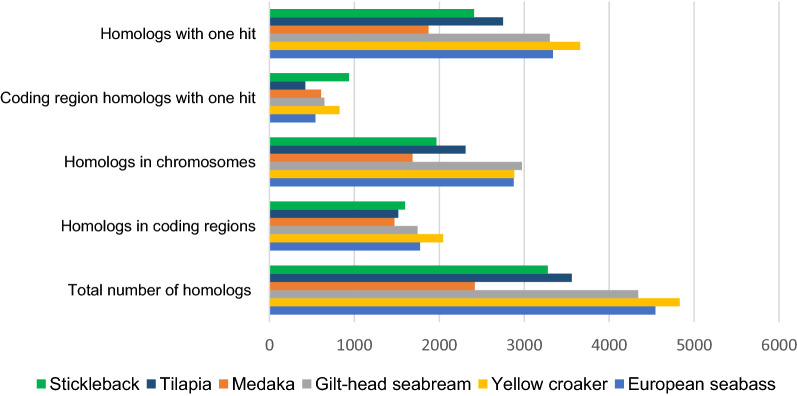
Quantitative summary of the comparative genomic analysis. The y-axis shows the homolog categorization depending on whether it is a BLASTN search, against the whole genome or just the coding regions and if the search only corresponds to one hit or multiple. While, on the x-axis is the corresponding count, in hits, for each of the six species. *Total number of homologs, corresponds to the totality of the 5988 SNP markers, while all the other categories, refer only to the 4529 SNP markers that were included in the genetic map.

The homologs identified that had a single hit against the six genomes were 2408, 2750, 1873, 3301, 3657, 3338 for stickleback, tilapia, medaka, gilthead seabream, yellow croaker and European seabass, respectively (Fig. 2). Yellow croaker showed, as expected, the highest homology with meagre, followed by European seabass and gilthead seabream, with medaka, tilapia, and stickleback showing the fewest homologies. Homologies in the coding regions with one hit, between meagre and the six species, resulted the highest against stickleback followed closely by yellow croaker. The percentage of each meagre LG, that was found homologous to a chromosome, shows the degree of synteny between meagre and each of the 6 species.

**Figure 3:**
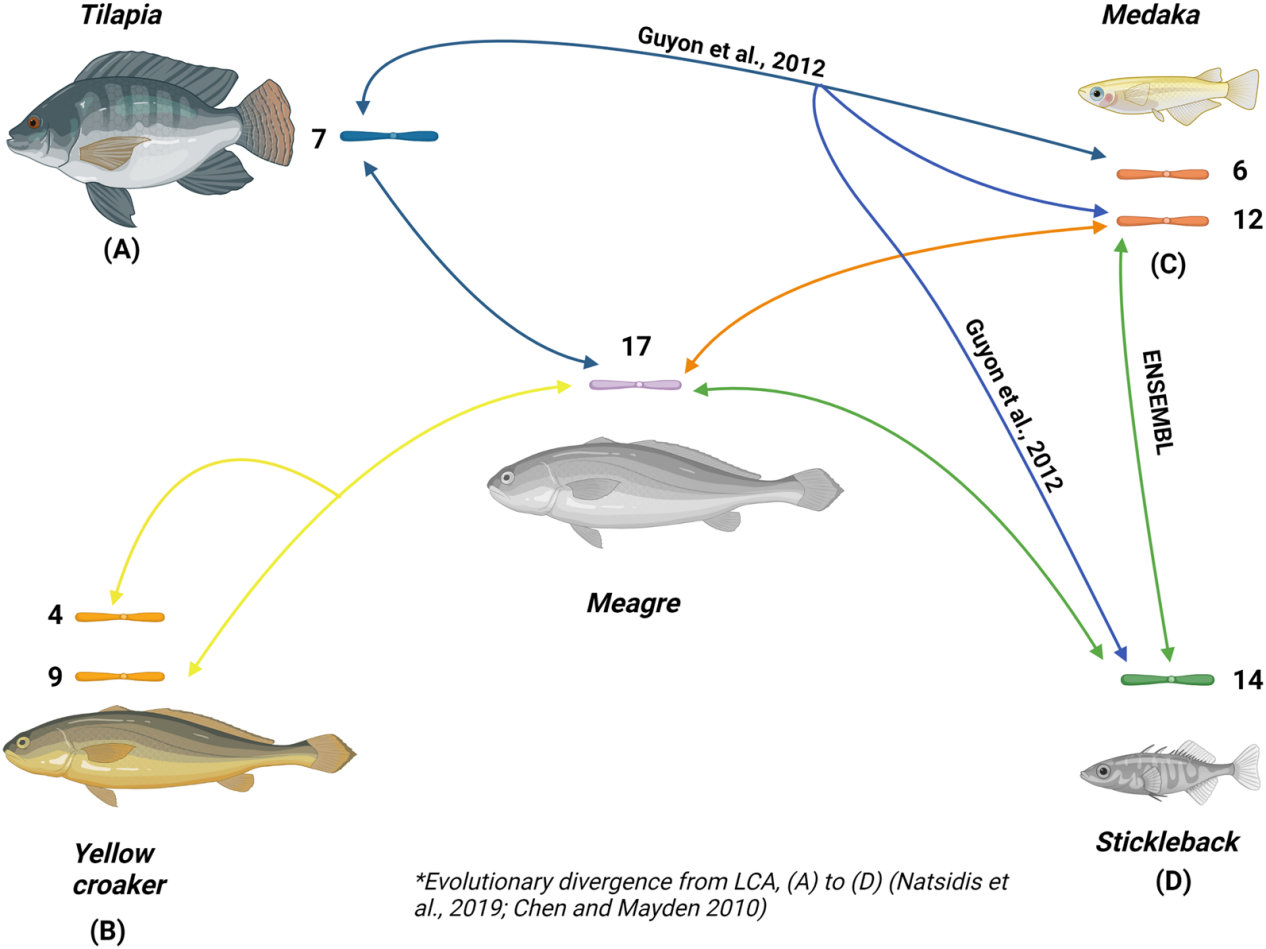
Meagre’s LG17 intricate homologies. (**A**)–(**D**) refers to the putative order of divergence from the last common ancestor^44,45^, showing a series of rearrangements^46^, ENSEMBL comparative genomics database, and the findings of this study.

The average percentage of homologous markers of the 24 meagre LGs are 76.4%, 80,4%, 90.4%, 90.6%, 86.1%, 92.4% for stickleback, tilapia, medaka, gilthead seabream, yellow croaker and European seabass, respectively (Fig. S1). European seabass showed the highest percentage of homologies along the meagre LGs with 92.4%. Medaka (90.4%), showed a disproportionately high percentage in relation to the count of homologies shown, being the second highest along with gilthead sea bream. The corresponding order of the meagre LGs and the homologous chromosomes of the 6 species is shown in Table S3. We built six Circos plots to visualize these homologies, each for every comparison. Firstly, Fig. S2 shows the syntenic relationships between yellow croaker and meagre, where it is observed that three meagre LGs (1, 10, 17) showed 1:2 homology with 3 pairs of yellow croaker chromosomes, 1–21, 5–24, 4–9 respectively, while the rest chromosomes showed 1:1 homology. Secondly, Fig. S3 reveals the homologies between stickleback and meagre. Six meagre LGs showed 2:1 homology with three stickleback chromosomes, while the remaining meagre LGs showed 1:1 homology with the rest stickleback chromosomes. Thirdly, Fig. S4 shows the homologies between meagre and European seabass. Meagre LG1 was found 1:2 homologous to seabass chromosomal pair 2–6, while the remaining chromosomes were found 1:1 homologous. Fourthly, Fig. S5 illustrates the high degree of homology between meagre and medaka, where meagre LG1 was found 1:2 homologous, to medaka chromosomal pair 6 -10, and the 22 remaining chromosomes where homologous in 1:1 fashion. Fifthly, the homologies between meagre and tilapia are shown in Fig. S6, where chromosome 7 of tilapia showed 1:2 homology with meagre LGs 1–17, and LG1 of meagre is homologous to tilapia 2–7, suggesting a possible fusion. The sixth comparison with the gilthead sea bream genome is visualized in Fig. S7 where, the only 1:2 correspondence was between meagre LG1 that was found homologous with gilthead sea bream’s chromosomal pair 8–18, with the remaining chromosomes between meagre and gilt-head seabream showing a high degree of 1:1 homology. Lastly, meagre LG17 which was found homologous to the yellow croaker chromosome pair 4–9, showed to be correlated with rearrangements in medaka (chr12) and tilapia genomes (chr7), with stickleback chr14 which in turn is homologous to both medaka chr12, tilapia chr7 and meagre LG17. Figure 3 depicts this network or homologies in a clear way.

### QTL mapping and gene ontology

Weight (kg) and total length (cm) were measured, in all 800 fish of the two batches, constituting the phenotypic data set. In the two batches, the mean values for length were 61.92 cm for batch 1 and 62.17 cm in batch 2 and for weight were 2.289 kg for batch 1 and 2.317 kg for batch 2^47^ (Supplementary data file 2).

**Figure 4:**
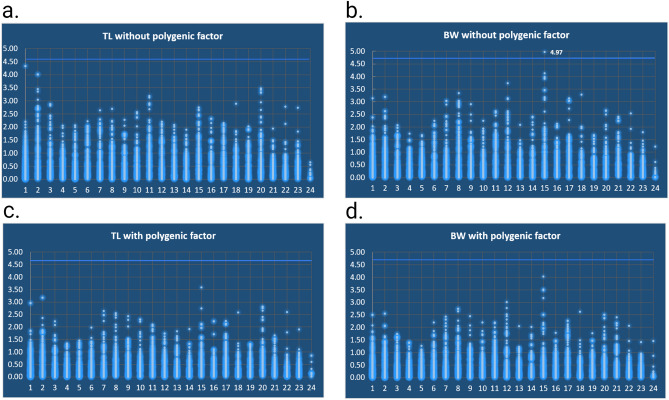
QTL scan results for the 2 models concerning BW (body weight) and TL (total length) traits. The x axis depicts the LGs from 1 to 24, and the y axis the LOD (logarithm of odds) scores that the markers attained for each model, trait and LG. The light blue lines are the threshold LOD values, which should be surpassed for a marker to be considered statistically significant.

Quantitative Trait Loci (QTL) mapping utilizing the newly developed genetic linkage map was performed for two phenotypic traits, Body Weight (BW) and Total Length (TL). The models implemented for the two traits were the polygenic and the non-polygenic, i.e. with and without the effect of the polygenic factor, respectively. The slight differences the LOD thresholds for polygenic and non-polygenic models show are attributed to the threshold estimation by perturbation-based simulations. Total Length (TL) did not show significant association with any marker (Fig. 4a,c). The highest LOD scores for this trait were estimated on LG1 (single SNP with no trailing LOD score from other SNP in the LG) and on LG2. However, when a polygenic component was included in the analysis model the highest LOD score was as in the case of BW, in LG15 (Fig. 4c).

The RAD locus “36880” which is situated in meagre LG15, seems to be significantly associated with a BW QTL with a LOD score of 4.97 (threshold 4.59) (Fig. 4b). This locus is found in coding areas of the the *eys* gene family. Locus “36880” is located at the intron that is between exons 42 and 43 (*A. regius* gene models, gene “G09265”, Papadogiannis et al. in prep), and specifically the locus starts 2 nucleotides after the stop codon of exon 42 at the 5′ UTR. The “G09265” gene has 24 domains that belong to the EGF superfamily (epidermal growth factor domains), which have been associated with growth traits in tilapia, cattle, and sheep^41^ (protein sequence in Supplementary data file 1). The variance explained by the locus and specifically the heterozygous marker genotype, symbolized by SB in Fig. S9, was 0.31 explaining 31% of the variance for the non-polygenic model, while the two homozygous marker genotypes SS, BB, were negatively affecting BW (− 0.16 and − 0.15 estimates, respectively). However, when the model of the QTL analysis included a polygenic component to account for any genetic effect from the rest of the genome on the trait (BW) the significance of the SNP effect disappeared (Fig. 4d).

## Discussion

In this study, we report the first genetic linkage map for meagre, *Argyrosomus regius,* along with the first QTL analysis investigating the genetic basis of two growth related traits, and the comparative genomic analysis with other species. The three constituents, shift from an introspective to a retrospective viewpoint, providing, with knowledge that give value to a rapidly emerging species in aquaculture.

Furthermore, the groundwork for the genetic basis of the weight trait at early life stages is laid, and interesting insights concerning the genomic make up of meagre, the estimated degree of conservation with well-known teleost, elements of karyotype evolution, and putative rearrangement events are inferred.

### Using ddRAD markers for the construction of genetic linkage maps

Recombination events in known crosses of aquaculture species, have been widely investigated using restriction-site associated DNA marker based genetic linkage maps^11^. The implementation previously described^26^, uses a ddRAD variant where the ligated DNA samples with the barcoded adapters are pooled together. The ddRAD could introduce errors into the analysis of teleost, due to a spurious distinction between paralogous and normal allelic SNPs deriving from ancestral genome duplication, such as in salmonids^46^, or mutations in restriction endonuclease recognition sites. However, the high coverage per sample used in this study, i.e. 43.3×, can overcome such hurdles. An advantage of using two restriction enzymes, in contrast to a single enzyme like in RAD sequencing, along with STACKS pipeline^42^ is that pair-ended Illumina sequences are utilized to increase the SNP identification accuracy. However, there is a trade-off as Stacks pipeline cannot distinguish between insertion/deletion fragments as it utilizes both P1 and P2 reads solely for the SNP calling procedure, whereas in *pyRAD*^48^, for instance, the P2 reads are used as a means to filter out putative insertion/deletion fragments. It is of importance to note, that insertion/deletion sequences can be a problem with a low sample number and a not so high sequencing coverage, which is not the case in this study. While genome re-sequencing could be an alternative and viable option for SNP calling^49^, it can be costly, especially for large genomes, making it a more cost-effective method for high resolution population genomic studies where tens of samples are used per population. The flexibility of ddRAD-seq and the competitive benefit it provides with the declining sequencing costs, make it still, a strong candidate for medium to high-density SNP identification purposes.

### Genetic linkage map

Traditionally, biparental mapping populations are produced from two inbred lines. Many software packages exist for map construction for such populations, like MapMAKER^50^, JoinMAP^51^, and CarthaGène^52^. Multi Parent Populations (MPPs) are genetically mixed populations deriving from a small set of known founders. Multiple founder alleles, originating from MPPs provide advantages over traditional two-parent crosses by producing higher resolution and more informative marker patterns that can help identify causal variants and distinguish pleiotropy from a random co-localization of multiple QTL^53^. Additionally, higher resolution mapping deriving from MPPs, can result in fewer candidate genes, and can minimize the confounding effects of linked loci. The Rqtl2^54^ was used in this study due to its capacity to produce genetic maps from MPPs. A genetic map can be affected by missing genotypes, especially if SNP imputation is not preferred^55^, and it has been shown by simulations that undetected typing errors at a rate of ~ 1% can lead to incorrect map ordering and inflation of LG lengths as marker density increases^56^. However, if the distances between the genetic markers are large, e.g., > 50 cM, the marker density may not be sufficient for an optimal representation of the genome. The greater the overlap between the genotypes of the offspring and the parents, the greater the accuracy of the chromosomal recombination ratios, and the less intervals in the map. The linkage map reported in the current study does not show long intervals in the LGs; however, it shows two ~ 20 cM intervals in LG1, which is also significantly longer than the rest (Fig. 1, Table S2). It is worth noting that the distribution of loci on the map is relatively uniform, except LG1 and LG24 (the longest and the shortest LGs, respectively), with most LGs having ~ 170–200 markers. Because the genetic map included RAD loci that were shared by 70% of the individuals, putatively missing genotypes played a role in the intervals showed in LG1. For this reason, a second genetic map was constructed by using a subset of RAD loci with greater coverage, and subsequently, less missing genotypes, so as to test whether the two gaps in LG1 would decrease, due to the increased resolution of the fractions of chromosomal recombination. Based on this hypothesis, a genetic map was created for a subset of 90% shared RAD loci, and the same MAF of 0.01, (Fig. S8), which indeed showed reduced gaps in LG1 by 30 cM but still the LG1 does not break into two. However, no statistically significant results were obtained from the QTL mapping analysis from the genetic map utilizing the 90% shared loci subset (Fig. S10). Therefore, the initial map with the 70% shared RAD loci was considered more optimal. However, the coverage of the ddRAD sites does not adequately explain the final configuration of the genetic map, which can putatively be attributed to a combination of factors. A centric fusion of two acrocentric chromosomes whose centromeres are located in the periphery results in the formation of a metacentric chromosome^57,58^. Conversely, a centric fission of a metacentric chromosome leads to the formation of two acrocentric chromosomes. It has been shown that in teleost fish, from a sample of 2587 karyotypes, ~ 30% of the species have karyotypes with acrocentric chromosomes exclusively^59^. In particular, the two large monophyletic groups, Eurypterygii and Otophysi showed differences in the distribution of chromosomal arms in their karyotypes. Euryptegygii include the species mentioned in this study and make up the majority of today's marine fish. A proportion of 45% of the 1368 karyotypes of Eurypterygii, appeared to be 100% comprised of acrocentric chromosomes. In addition, it was shown^59^ that the rate of central fusion/fission depends on the number of initial acrocentric and metacentric chromosomes, the acrocentric chromosomes for fusion and the metacentric chromosomes for cleavage, showing that there is a link between chromosome evolution and the number of arms. Based on the above observations, it is possible that the meagre karyotype has a metacentric chromosome (LG1) and 23 acrocentric chromosomes. While the data resulted to the best possible chromosome configuration, one alternate hypothesis could also include parts of LG1 grouping with the remaining chromosomes or even with a sex chromosome, and this was not possible to observe based on the given chromosomal recombination event ratios between parents and offspring. Lastly, the two sex-specific maps constructed, did not reveal a sex-bias in the clustering of the markers in the LGs, nor a noticeable difference between them in total length (less than 5%, 1899 cM and the female one 1987 cM). The slight difference in total length can be putatively attributed to the higher number of male to female breeders, i.e. 4:3 respectively.

### Comparative analysis

The comparative genomic analysis, between meagre and six teleost species, showed overall a high conservation of synteny of the ancestral 24 chromosome karyotype of meagre. Especially, the high percentage of participation of meagre LGs in homologous chromosomes in a 1:1 pattern, as well as the high number of unique blast hits, indirectly verified the overall high quality of the genetic map, and hinted on the high quality of the genome assembly. Particularly, the high degree of synteny observed between the medaka and meagre, agreed with the same comparison between the yellow croaker (which is also a Sciaenid), and medaka^31^. The evolutionary proximity between Sciaenids and European seabass, which was shown^44^ using the yellow croaker genome, was also reflected in this study, showing the same pattern with European seabass being phylogenetically closer to meagre than gilthead seabream. Moreover, the same 1:2 chromosomal homologies between stickleback-meagre, and stickleback-European seabass were shown. Specifically, the fusions in stickleback chromosomes 1, 4, 7 corresponded to meagre LG pairs 3–22, 19–1, 14–23 which corresponded to seabass (*Dicentrarchus labrax*) chromosomal pairs 13–14, ×–2, 14–23 (in 1:1 homology), indirectly agreeing with previous findings^60^, concerning the stickleback and seabass comparison (Table S3). Another consistent result was the higher coding homologs identified between stickleback and meagre, relative to the other teleosts of this study, which is also observed in other studies between stickleback and yellow croaker^31^, and stickleback and medaka^61^. This, could putatively be attributed to the very high quality of the stickleback genome assembly in comparison to the other teleost. A putatively prominent chromosomal rearrangement (fusion), was observed between meagre and yellow croaker through the 1:2 homology of meagre LG17 and yellow croaker chromosome pair 4–9. The LG17 in meagre was found homologous to stickleback chr14, medaka chr12 and tilapia chr7, which in their turn were deemed homologous by^46^, and the ENSEMBL comparative genomics database. Yellow croaker, showed high degree of homology with meagre but not the highest, which was the expected result. In^31^, RNA-seq was used for the construction of the yellow croaker genetic linkage map, which shows a high degree of synteny with medaka, and in the present study the degree of synteny of meagre with medaka follows the same pattern. The difference between the two studies, is that the genetic map of meagre is comprised both by coding and non-coding sequences while the yellow croaker map just by coding sequences. Therefore, the high synteny observed in^31^, does not entail repetitive regions in the yellow croaker genome which are error prone in homology inference among phylogenetically close species. Furthermore, the coverage of^31^ was 3.5% greater than 95.8%, which was the coverage achieved in the study of the yellow croaker genome^62^ in the assessment of the completeness of the assembly with 18,000 transcripts. Although a comparative genomic assay that detects homologies in coding and non-coding regions is more likely to detect chromosomal rearrangements, this is not definite as a number of parameters may affect the outcomes such as sequencing depth, reads quality, number of sequences relative to genome size and polyploidy.

### QTL analysis

It is widely accepted that the genetic basis of quantitative traits, such as body weight and length, is mostly due not to single gene effects, but to the causal interaction of groups of genes^63,64^. The results presented in this study detected a statistically significant QTL for weight without the effect of the polygenic factor, and ontologically identified it as the *eys* gene. This finding, is an incomplete representation of the additive genetic variance that explains weight as a phenotypic trait, however, it is highly likely that it is indeed part of the broader genetic basis of the weight trait of meagre, as it was shown to play a role in growth, in a series of domesticated vertebrates, with tilapia among them.

Nevertheless, the findings of the second growth related quantitative trait (i.e. TL) could potentially explain these hypotheses^63,64^. However, further investigation is needed for any possible interaction of candidate genes in LG15 (such as the *eys* gene) and in LG1 and or LG2, where Total Length expressed the highest LOD score.

## Methods

### Sample selection, DNA extraction, microsatellite genotyping and parentage assignment

The fish used in this study belong to the first batch, and were measured for length and weight (see^47^ for details). The separation in two batches, one with the smaller juveniles and one with the larger ones, is a standard practice in commercial conditions from fish originating from the same spawning event, so as to avoid cannibalism in the early growth stages. The measurements were made for the first batch (400 fish) in January 2016 and for the second batch (400 fish) in May 2016, i.e. when the fish reached the commercial weight of approximately 2 kg. Six families comprising of 266 samples that showed high phenotypic variation in weight and length were selected. These families comprise four male, three female breeders and their offspring. DNA was extracted from all fish using standard protocols^65^ and DNA quality and quantity was evaluated using a NanoDrop ND 1000 spectrophotometer (Thermo Fisher Scientific). All fish were genotyped using a ten microsatellite loci multiplex^66^, and the families that were found in^47^ were used, while parentage assignment was done according to^67^.

### Construction and sequencing of the ddRAD libraries

After the DNA extraction, an RNAse treatment for all 266 samples was followed. DNA was first eluted in 5 mmol/L Tris, pH 8.5, and stored at 4 °C. Each sample was quantified by the Nanodrop 1000-Thermo Fisher Scientific and a 0.7% agarose gel was used for quality assessment. For the ddRAD library construction, an already described protocol^27^ was used. In each of the 266 DNA samples (7 parents in triplicate and 259 offspring; 15 ng DNA per sample), separate digestion was performed, using the same incubation time and synchronously, of the two high-fidelity (RE) restriction enzymes: *Sbf*I (CCTGCA|recognition site GG) and *Nla*III (CATG|recognition site C), (New England Biolabs, NEB, UK). Digestions were incubated at 37 °C for 90 min, using 20 U of enzyme per microgram of DNA and 0.6 μl of CutSmart Buffer (NEB), at a total reaction volume of 6 μl. The reactions were let to cool at room temperature, and 3 μl of an adapter mixture was added, and incubated at room temperature for 10 min. The adapter mixture contained individual combinations of P1 (*Sbf*I-compatible) and P2 (*Nla*III-compatible), at concentrations of 6 and 96 nM, respectively, in a 1 × reaction buffer No. 2 (NEB). The ratio of adapter P1 to P2 (1:16) was chosen in accordance with the relative abundance of *Sbf*I and *Nla*III cutting sites. For sample identification after the sequencing step, adapter P1 and P2 included a five to seven-base sequence. Ligations were performed over 3 h at 22 °C by adding 3 μl of ligation mixture of 4 mM rATP (Promega, UK) and 2000 T4 ligase units (NEBs) in a 1 × CutSmart buffer (NEB). Pooling of the ligated samples was performed followed by a column purification step (MinElute PCR PurificationKit, Qiagen, UK), and elution in 70 μl EB buffer (Qiagen, UK). Size selection of the ligated pooled samples was performed by agarose gel separation, using a selection window between 450 and 750 bp. A gel purification step followed (MinElute agarose gel extraction kit, Qiagen, UK), and selected DNA fragments were eluted (68 μl in EB buffer). PCR amplification was performed on the size-selected fragments (15 cycles of PCR; 32 separate 12.5 μl reactions, each with 1 μl Template DNA) using Taq high-fidelity polymerase (Q5 Hot Start High-Fidelity DNA Polymerase, NEB). After the pooling of the PCR reactions (400 μl in total), purification followed by a column (MinElute PCR purification kit). The 57 μl eluate in EB buffer, was then further purified using an equal volume of AMPure magnetic beads (Perkin-Elmer, UK) to maximize the removal of small fragments. The library was eluted in 24 μl EB buffer and sequenced in a HiSeq4000 (2 × 150 bp).

### Quality control, genotype calling and loci filtering with STACKS pipeline

STACKS pipeline^42^ was performed in the snakemake-based singularity container built previously^68^. Briefly, we used FastQC v.0.11.5 software to perform quality control of the primary sequencing data^69^. To retrieve the reads that correspond to each individual, we filtered and separated the sequences using the process radtags module from the STACKS v.2.3 pipeline^42^. In this step, the − q parameter was used to filter out low quality sequences (below 20) using the Phred score provided by the FASTQ files. BWA software^70^ with default parameters was used to align the sequences to the in-house reference genome of *Argyrosomus regius* (Papadogiannis et al., in preparation). The genome of mulloway *A. japonicus*^71^ which is comprised of 24 chromosomes based on Hi-C data^71^, was used as the linkage group reference number for the *A. regius* genetic map construction. gstacks module was used to create the ddRAD loci and finally, populations module of STACKS was used for the final filtering of the loci for a subset of 70% of the ddRAD sites among the 266 samples, − r 70, and − maf 0.01 (minor allele frequency). Furthermore, an additional subset of SNPs was chosen with − r 90 and − maf 0.01, using the exact same procedure, for the evaluation of the genetic map construction, the linkage groups length, and the marker intervals.

### Quality control with Plink

Plink1.9^72^ was used with the parameter − geno 0.3, which is the equivalent of − r 70 of STACKS, as well as the parameter − mendel to find genotyping errors between parents and offspring that cannot be explained by Mendelian inheritance. The permissible errors per site were set at 5 or 2%.

### Construction of the genetic linkage map

LepMap2 software was used to construct the genetic map^73^. The SNPs that passed the Data Tolerance threshold (0.001), were 4529 out of 5,988 and were distributed to the Linkage groups using LOD score 4.80, so that the number of LGs generated was equal to the number of chromosomes set at n = 24. Commands were used as in^42^, which can also be found in their default configuration in Java script format in LepMap2 package. By repeating the same procedure, four maps were created in total. Two, utilizing the two different SNP marker subsets, of 70% and 90% shared loci per sample, and two sex-specific maps, for males and females. The three additional maps were created. The visualization of the genetic linkage map was done with LinkageMapView^74^.

### Comparative genomics

All RAD loci included in the meagre LGs were used in comparative analysis against genomes from the following teleost genomes: medaka (*Oryzias latipes*, Ensembl 100), Nile tilapia (*Oreochromis niloticus*, ncbiGCA_001858045.3), European sea bass (*Dicentrarchus labrax*, v1.0c http://seabass.mpipz.de), stickleback (*Gasterosteus aculeatus*, ncbiGCA_016920845.1) gilthead sea bream (*Sparus aurata*, Ensembl 100), and yellow croaker (*Larimicthys crocea*, Ensembl 100). Nucleotide sequences of ddRAD markers included in the meagre´s 24 LGs were used in a BLASTN search to find homologous regions in the respective five aforementioned genomes. The e value limit was set at 10^–9^ and results with more than 10 hits or more than 10 HSP (high scoring segment pairs) in the first scoring match were excluded to eliminate duplicate regions in the genome. Only the top hit was kept, and was considered homologous to the target genome. The correspondence of the meagre linkage groups with the chromosomes of the target genomes of the 6 fish was deduced from this process. If the majority of meagre sequences from a single LG were found in homologous regions on one of the target chromosomes, then that particular meagre LG and that particular target chromosome were considered homologous. The exact same procedure was performed for the coding areas. The coding sequences of the 6 genomes were used in a second round of BLASTN analysis, with the same parameters, to investigate the homology of the genetic map exclusively with coding regions. The homologies of meagre LGs and the 6 genomes were visualized with Circos software^75^.

### QTL analysis

The genetic map in combination with the phenotypic characters were used to detect genetic regions related to the two phenotypes, body weight and total length. The analysis was performed with rqtl2 software^54^ using two models, with and without the effect of the polygenic factor, with the batch used as a fixed effect. The limit for the permutation test was set at 1000. Finally, the Logarithm of odds (LOD) significance thresholds for the model without the effect of the polygenic factor were 4.77 and 4.59 for body weight and length, respectively, while for the model with the effect of the polygenic factor were 4.76 and 4.58 for body weight and for length, respectively.

### Ethics statement

All experiments were performed in accordance with the “Guidelines for the treatment of animals in behavioural research and teaching” (Animal Behavior Society 2001). Sample providers complied with institutional, national, and international guidelines and regulations as well as Nagoya protocol to obtain our fish clip samples. No ethic committee approval was necessary for the collection of fish clips. All fish treatments used for sampling were in accordance with the guidelines of the European Directive (2010/63/ EU) on the protection of animals used for scientific purposes. In addition, *A. regius* is neither an endangered species nor a species at risk of Extinction according to the IUCN (Red List category: Least Concern).

## Conclusions

This study provides the first high-density genetic linkage map for meagre utilizing a multi-parental approach, as a valuable tool that can boost the aquaculture production of the species, help us study genome evolution, and improve the genome assembly.

The comparative genomic analysis, showed high level of consistency with previous studies, validated the integrity of the genetic map, shed light on events on the genome evolution of meagre, and characterized a putatively prominent fusion concerning linkage group 17.

For the first time, QTL mapping for early body weight provided a statistically significant result for this species, and the QTL seems to be mapped to the genic sequence of *eys* gene.

## Supplementary Information

Supplementary Information 1.

Supplementary Information 2.
