# Supplementary material for: Linkage mapping, comparative genome analysis, and QTL detection for growth in a non-model teleost, the meagre Argyrosomus regius, using ddRAD sequencing
Source: Sci Rep. 2022 Mar 29;12:5301. doi: 10.1038/s41598-022-09289-4 (PMC8964699; doi:10.1038/s41598-022-09289-4)
Supplement: Supplementary file 2 — Supplementary Information 2. [file 41598_2022_9289_MOESM2_ESM.docx]

**Supplementary material**

**Table S1. Summary of the two sex-averaged genetic maps**

**Table S2. Summary of the consensus genetic map of meagre**

| LG | marker number | average marker interval (cM) | length (cM) |
| --- | --- | --- | --- |
| 1 | 396 | 0.56 | 222.415 |
| 2 | 218 | 0.37 | 82.417 |
| 3 | 221 | 0.37 | 82.003 |
| 4 | 208 | 0.34 | 71.361 |
| 5 | 218 | 0.33 | 72.767 |
| 6 | 207 | 0.38 | 79.57 |
| 7 | 215 | 0.35 | 77.159 |
| 8 | 208 | 0.39 | 82.911 |
| 9 | 205 | 0.34 | 70.251 |
| 10 | 199 | 0.4 | 80.137 |
| 11 | 191 | 0.4 | 78.158 |
| 12 | 189 | 0.4 | 77.025 |
| 13 | 194 | 0.38 | 74.192 |
| 14 | 182 | 0.41 | 74.915 |
| 15 | 182 | 0.36 | 66.145 |
| 16 | 182 | 0.39 | 72.286 |
| 17 | 171 | 0.36 | 62.38 |
| 18 | 172 | 0.37 | 64.156 |
| 19 | 166 | 0.45 | 76.145 |
| 20 | 165 | 0.42 | 70.594 |
| 21 | 154 | 0.51 | 79.321 |
| 22 | 138 | 0.49 | 68.02 |
| 23 | 128 | 0.55 | 70.623 |
| 24 | 20 | 1.95 | 37.223 |

**Table S3. Homologies between the LG’s of meagre genetic map and the chromosomes of the 6 teleost species. Green colour depicts the chromosomes that are ordered the same as in the genetic map**

| **Meagre** | **European seabass** | **Yellow croaker** | **Gilthead bream** | **Stickleback** | **Medaka** | **Tilapia** |
| --- | --- | --- | --- | --- | --- | --- |
| 1 | LG2/LG6 | CHR1/CHR21 | CHR08/CHR18 | CHR4/CHR1/CHR20 | CHR6/CHR10 | CHR2/CHR7 |
| 2 | LG7 | CHR10 | CHR1 | CHR9 | CHR1 | CHR6 |
| 3 | LG13 | CHR7 | CHR2 | CHR1 | CHR13 | CHR14 |
| 4 | LG8 | CHR12 | CHR23 | CHR11 | CHR8 | CHR4 |
| 5 | LG1A | CHR6 | CHR6 | CHR18 | CHR5 | CHR5 |
| 6 | LG20 | CHR3 | CHR5 | CHR13 | CHR9 | CHR12 |
| 7 | LG5 | CHR8 | CHR4 | CHR2 | CHR3 | CHR1 |
| 8 | LG15 | CHR18 | CHR9 | CHR17 | CHR21 | CHR22 |
| 9 | LG22/25 | CHR15 | CHR7 | CHR12 | CHR7 | CHR19 |
| 10 | LG12 | CHR5/CHR24 | CHR16 | CHR15 | CHR22 | CHR18 |
| 11 | LG10 | CHR2 | CHR21 | CHR3 | CHR17 | CHR17 |
| 12 | LG18/21 | CHR23 | CHR19 | CHRY | CHR20 | CHR9 |
| 13 | LG2 | CHR11 | CHR22 | CHR19 | CHR24 | CHR15 |
| 14 | LG14 | CHR22 | CHR13 | CHR7 | CHR14 | CHR10 |
| 15 | LG11 | N/A | CHR15 | CHR6 | CHR15 | CHR13 |
| 16 | LG16 | CHR13 | CHR17 | CHR21 | CHR16 | CHR11 |
| 17 | LG19 | CHR4/CHR9 | CHR12 | CHR14 | CHR12 | CHR7 |
| 18 | LG4 | CHR17 | CHR11 | CHR8 | CHR4 | CHR16 |
| 19 | LGx | CHR20 | CHR14 | CHR4 | CHR23 | CHR16 |
| 20 | LG9 | CHR16 | CHR3 | CHR10 | CHR11 | CHR20 |
| 21 | LG1B | CHR19 | CHR20 | CHR5 | CHR19 | CHR8 |
| 22 | LG24 | CHR14 | CHR24 | CHR1 | CHR2 | CHR21 |
| 23 | LG3 | N/A | CHR10 | CHR7 | CHR18 | CHR3 |
| 24 | N/A | N/A | N/A | N/A | N/A | N/A |

**Figure S1. The number of markers per meagre LG that found homologies in each of the 6 species**


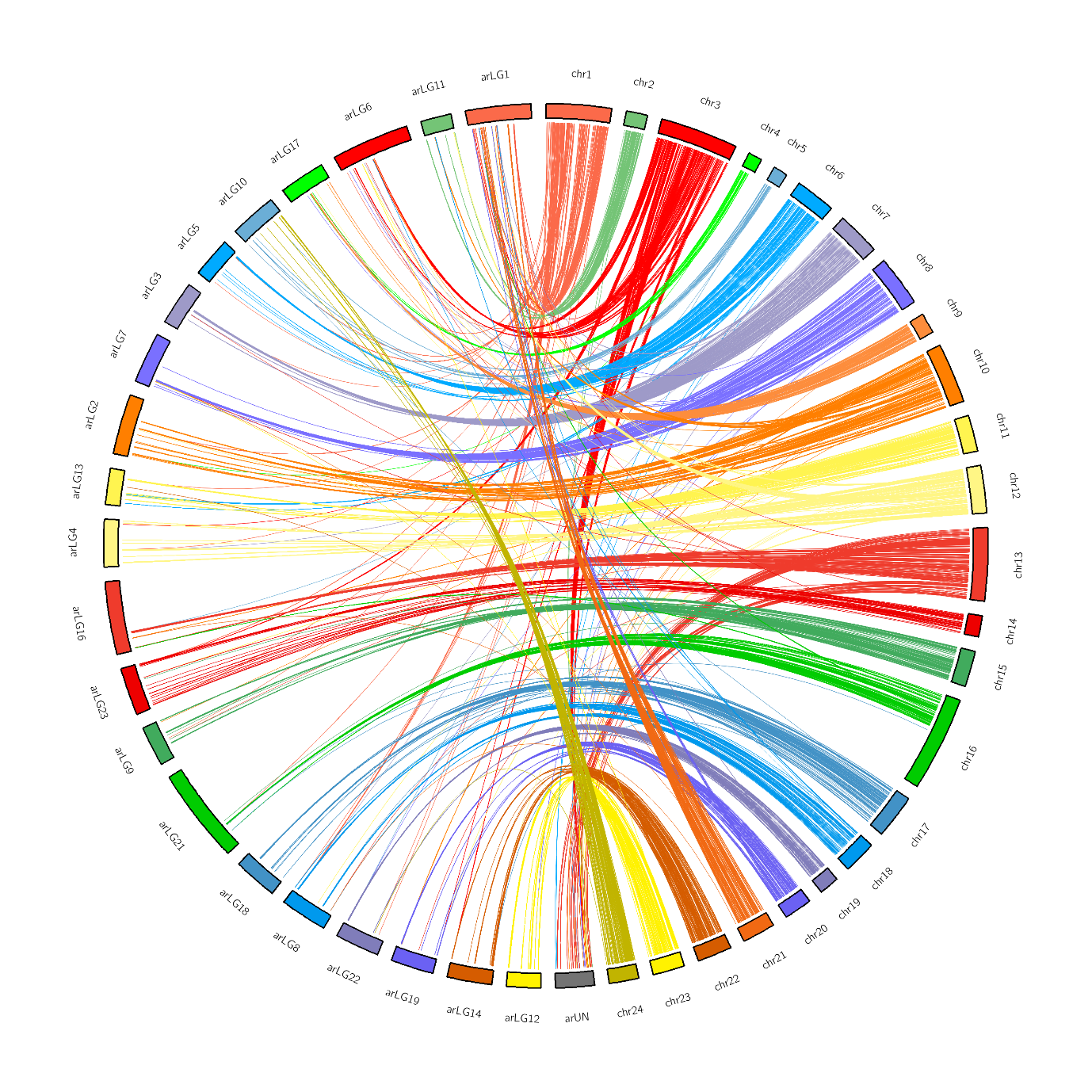


**Figure S2. Synteny between meagre and yellow croaker (24 chromosomes)**. The left semi-circle depicts the inferred homologous LG’s of meagre and the right semi-circle the chromosomes of yellow croaker in their natural ordering. ar_UN stands for,excluded from the genetic map, meagre markers that were found homologous with yellow croaker chromosomes


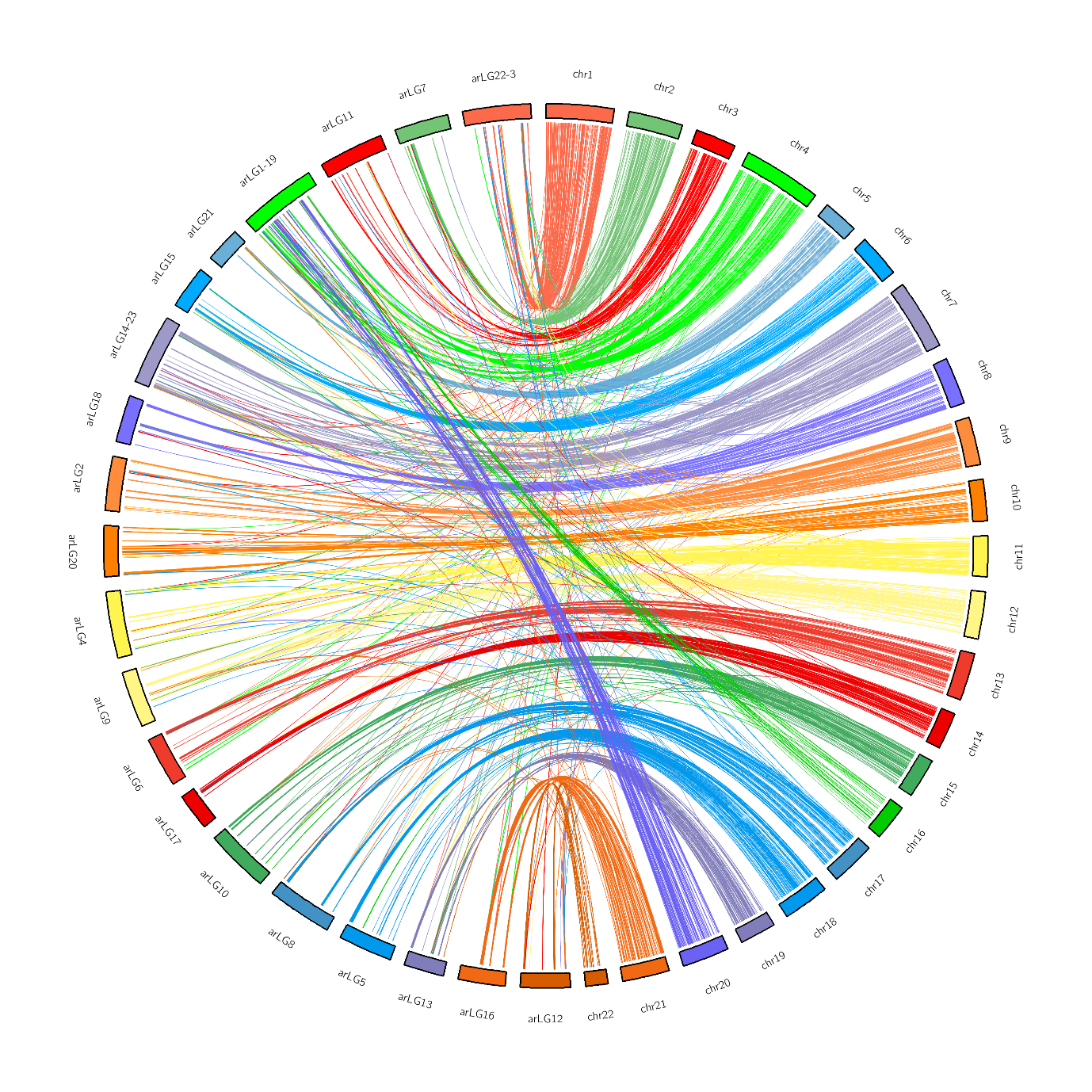


**Figure S3. Synteny between meagre and stickleback (21 chromosomes).** The left semi-circle depicts the inferred homologous LG’s of meagre and the right semi-circle the chromosomes of stickleback in their natural ordering (the 22^nd^ stickleback chromosome is the Y chromosome)


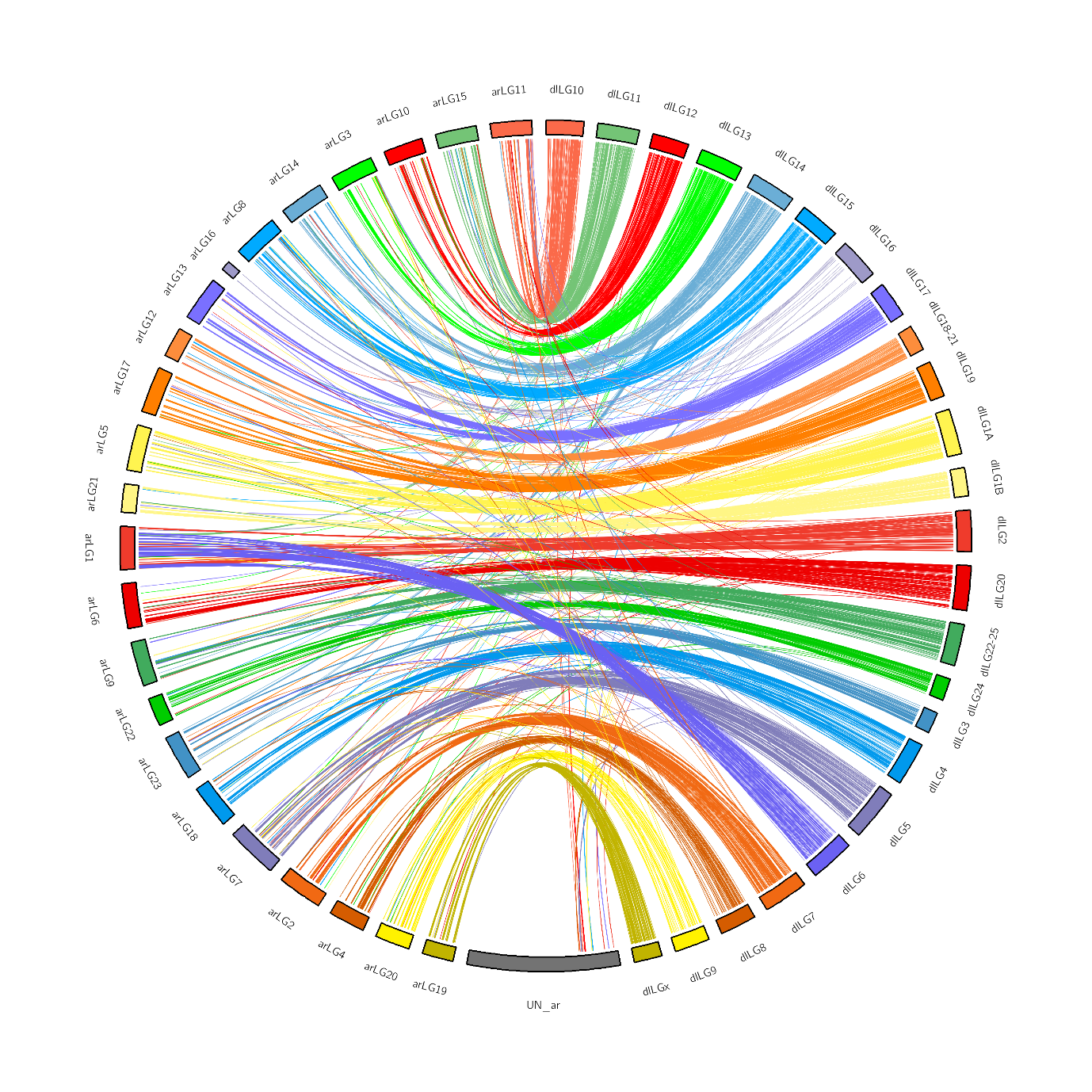


**Figure S4. Synteny between meagre and European seabass (24 chromosomes).** The left semi-circle depicts the inferred homologous LG’s of meagre and the right semi-circle the chromosomes of European seabass in their natural ordering. UN_arstands for the meagre markers that were found homologous with unassembledEuropean seabass contigs


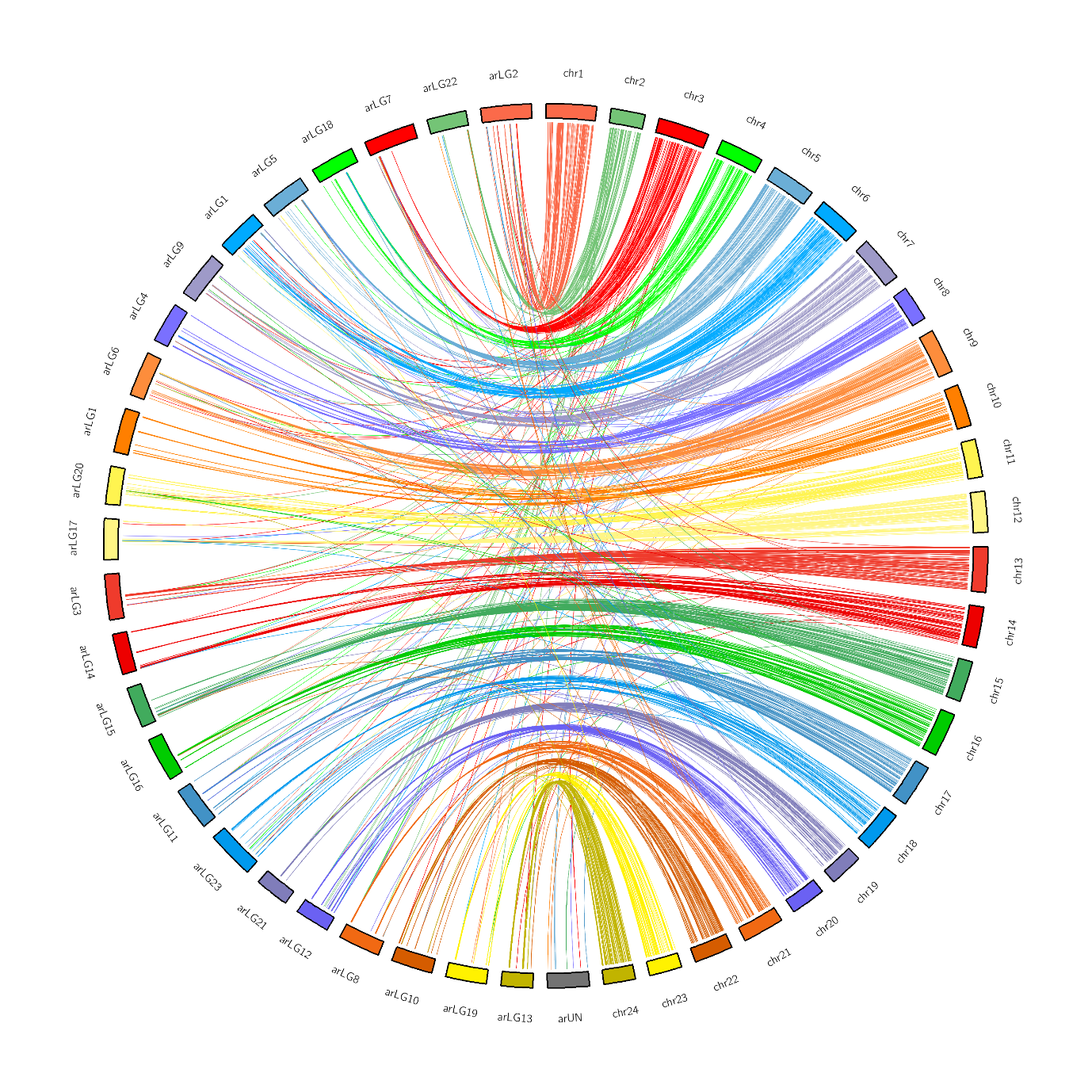


**Figure S5. Synteny between meagre and medaka (24 chromosomes).** The left semi-circle depicts the inferred homologous LG’s of meagre and the right semi-circle the chromosomes of medaka in their natural ordering. ar_UN stands for meagre homologies of sequences that were not included in the genetic map


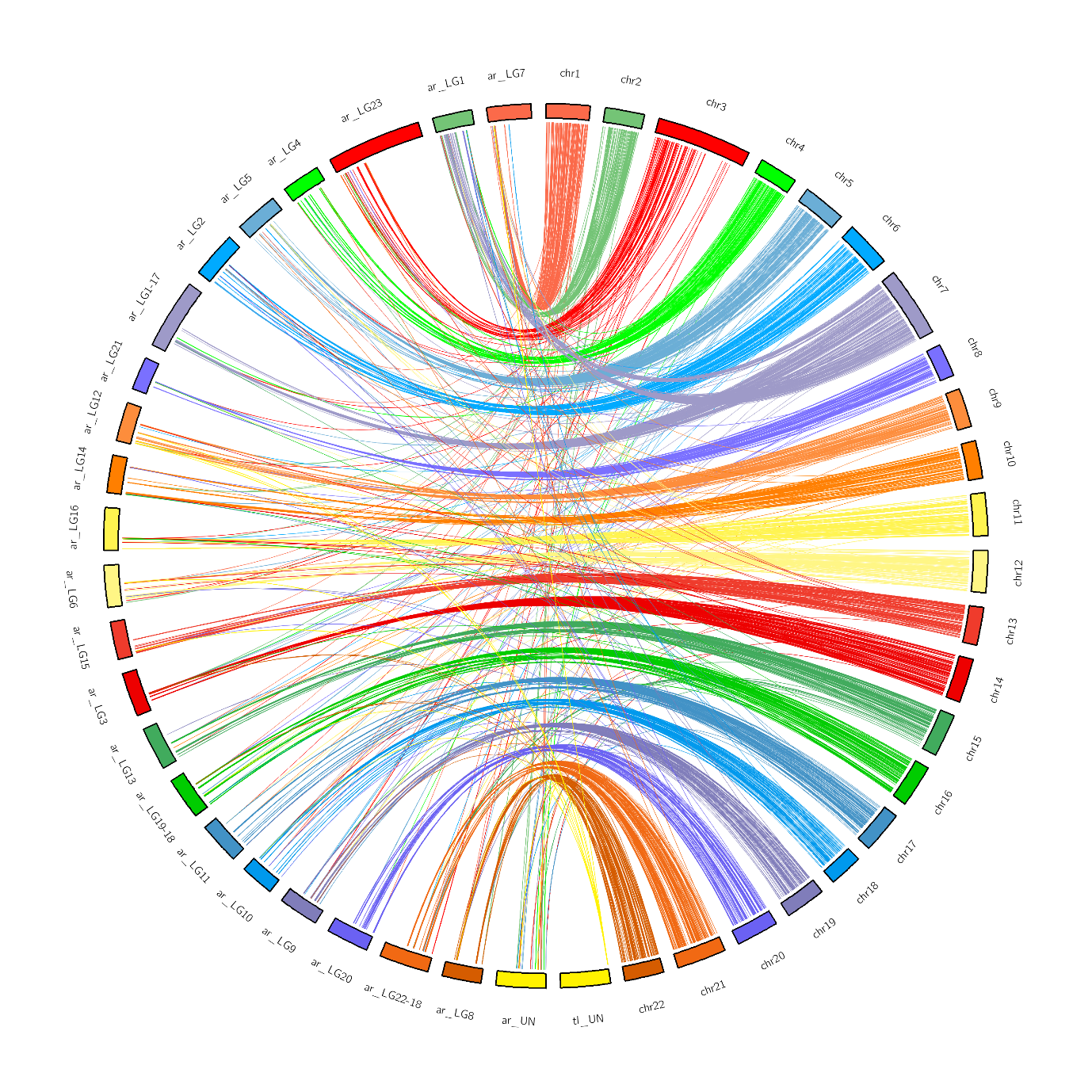


**Figure S6. Synteny between meagre and tilapia (23 chromosomes).** The left semi-circle depicts the inferred homologous LG’s of meagre and the right semi-circle the chromosomes of tilapia in their natural ordering. With ar_UN referring to homologies of loci not included in the genetic map, and tl_UN refers to loci of the meagre genetic map that found homologies with unassembled tilapia contigs


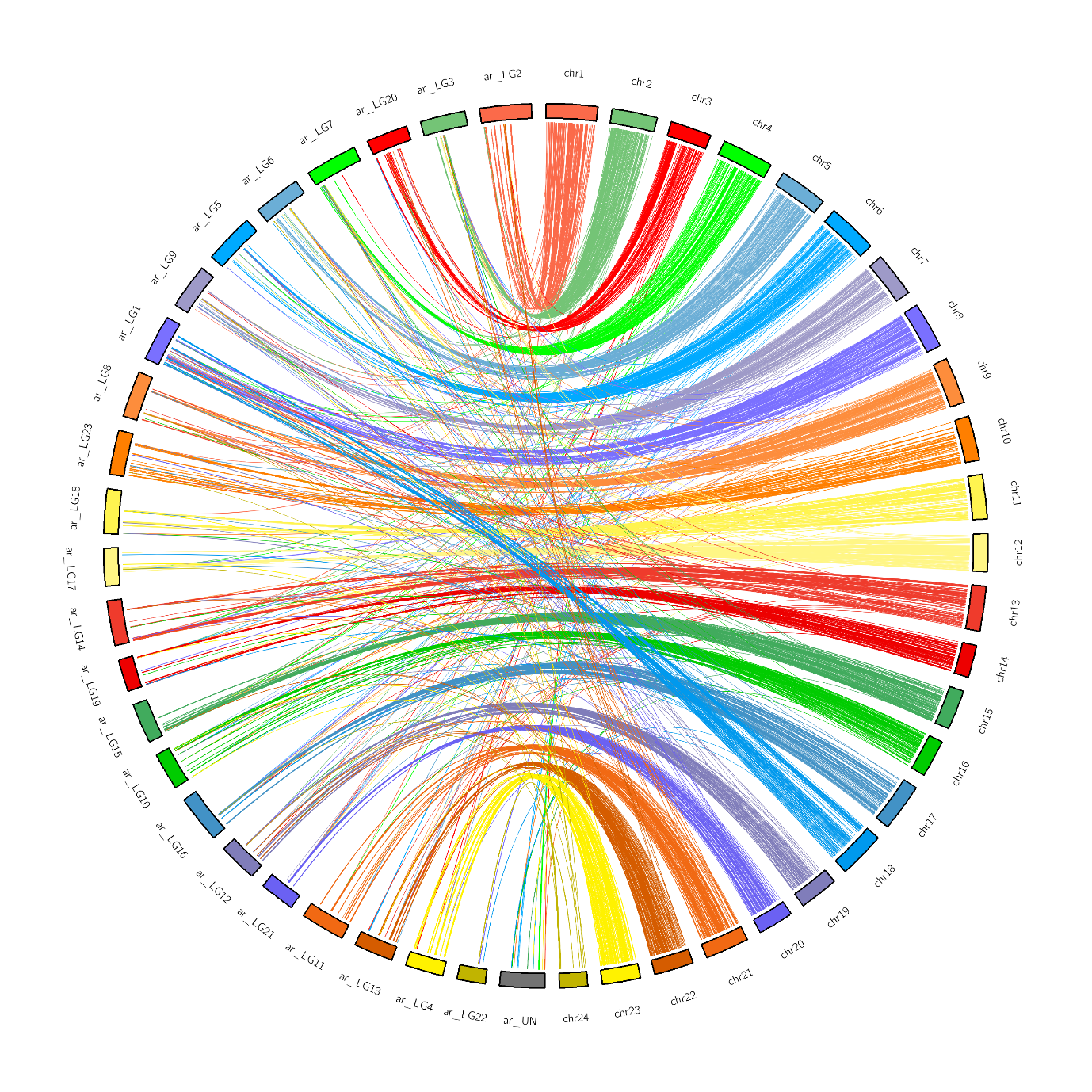


**Figure S7. Synteny between meagre and gilthead seabream (24 chromosomes).** The left semi-circle depicts the inferred homologous LG’s of meagre and the right semi-circle the chromosomes of seabream in their natural ordering. ar_UN refers to meagre homologies of sequences not included in the genetic map


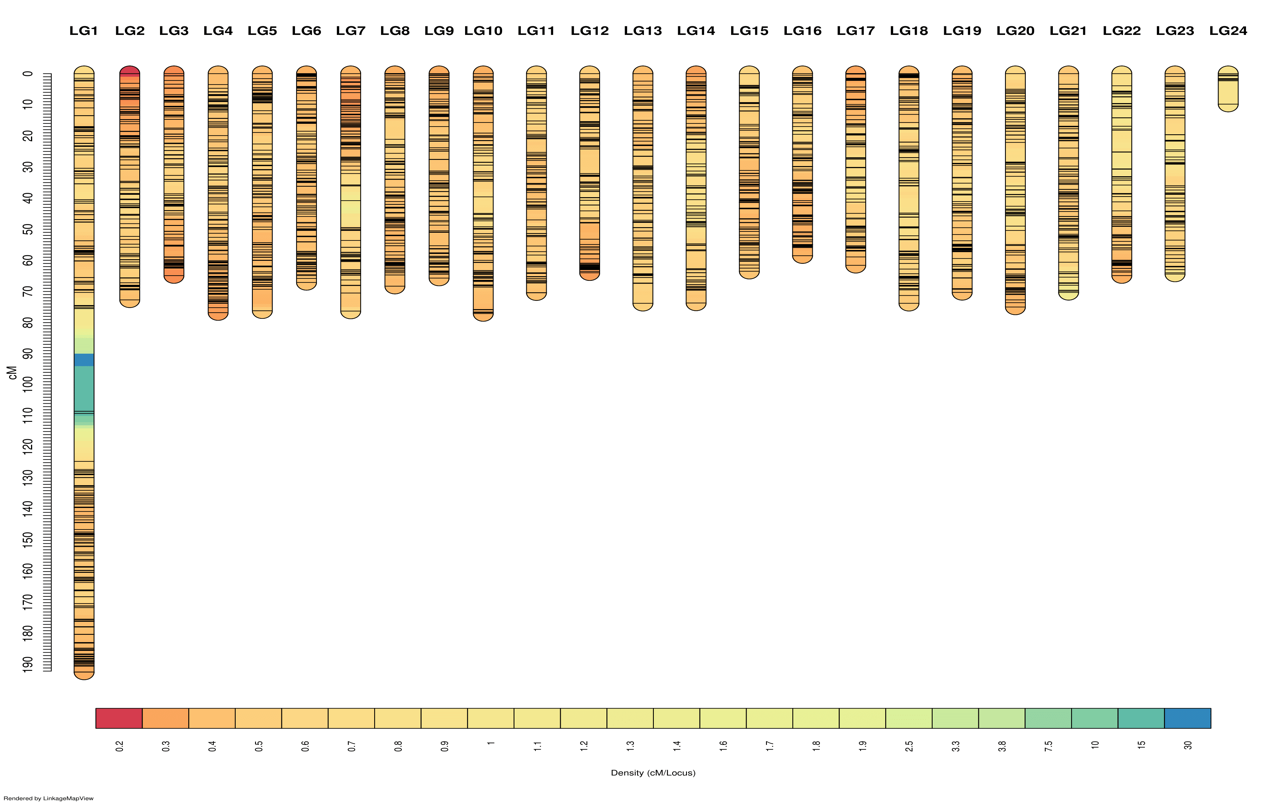


**Figure S8. Genetic map originating from a subset of 90% shared RAD loci among individuals**. The coloring of the marker intervals corresponds to a decrease in density, from red to blue. The 2 intervals of ~ 20 cM in LG1 decrease along with the subsequent length of LG1 by 30 cM compared to the one in Figure 1


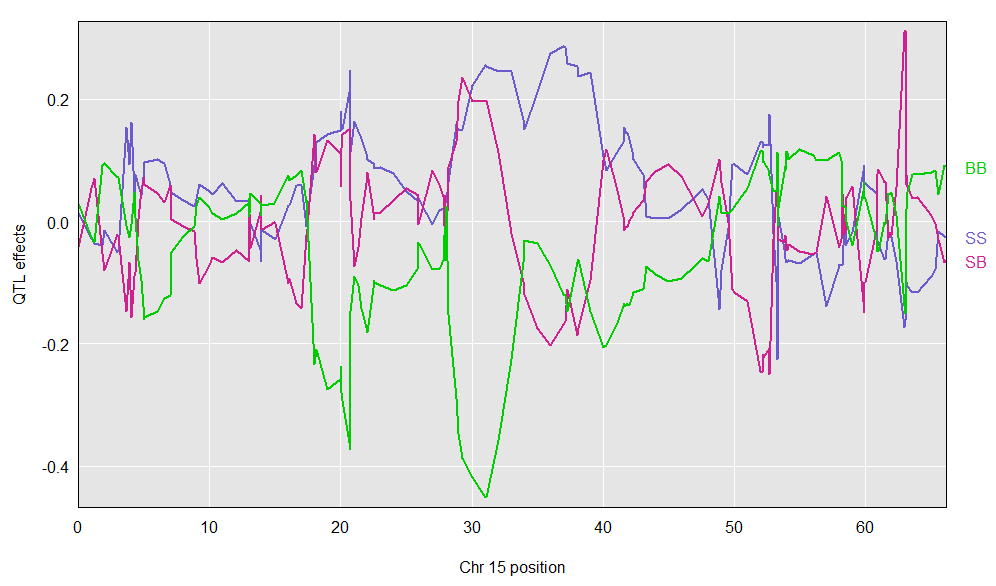


**Figure S9. Explained variance for the BW QTL of the weight trait without the polygenic factor.** SS, SB, BB stand for the biallelic genotypes of the S and B alleles. The explained variance of the genotypes associated with the 36880 locus that was found statistically significant were: SS -0.16148, SB 0.312388, BB -0.150912


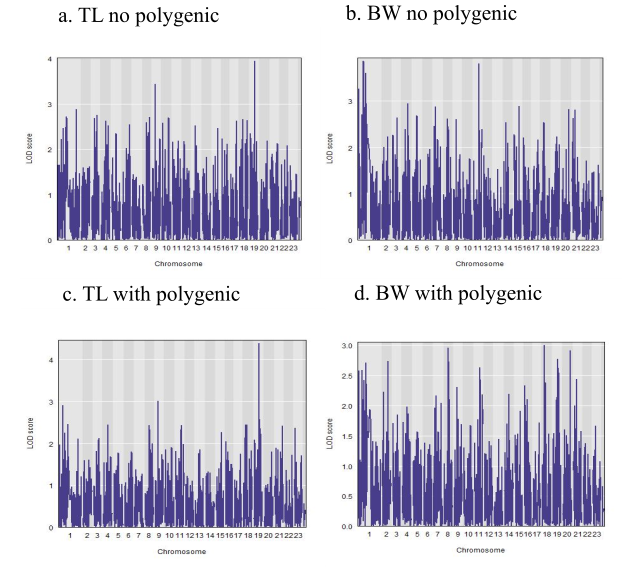


**Figure S10. QTL scan results for the 2 models concerning BW (body weight) and TL (total length) traits of the map with 90% shared RAD loci**. The x axis depicts the LGs from 1-24, and the y axis the LOD (logarithm of odds) scores that the markers attained for each model, trait and LG. The light blue lines are the threshold LOD values, which should be surpassed for a marker to be considered statistically significant
